# Recent and Cumulative Cannabis Use and Sleep in Middle-Aged Adults

**DOI:** 10.1001/jamanetworkopen.2026.38303

**Published:** 2026-10-09

**Authors:** Thanh-Huyen T. Vu, Kristen L. Knutson, Reto Auer, Julian Jakob, Donald M. Lloyd-Jones, Mercedes R. Carnethon

**Affiliations:** 1 Department of Preventive Medicine, Feinberg School of Medicine, Northwestern University, Chicago, Illinois; 2 Department of Neurology, Feinberg School of Medicine, Northwestern University, Chicago, Illinois; 3 Bern Institute of Primary Health Care, University of Bern, Bern, Switzerland; 4 Department of Medicine, Boston University Chobanian & Avedisian School of Medicine, Boston, Massachusetts

## Abstract

**Importance:**

Cannabis is widely used and often promoted as a sleep aid, but evidence of its long-term implications for sleep is limited.

**Objective:**

To examine the associations of cumulative cannabis exposure from young adulthood to middle age and recent cannabis use with objective and subjective sleep outcomes in middle age.

**Design, Setting, and Participants:**

This cohort study analyzed data from the Coronary Artery Risk Development in Young Adults (CARDIA) study, including 35 years of follow-up through the year-35 examination (conducted in 2020 to 2022), and the CARDIA Year-35 Sleep Ancillary Study. Analyses were conducted from January 15 to July 15, 2026.

**Exposures:**

Cumulative cannabis use (calculated in cannabis-years, with 1 cannabis-year equating to 365 days of use) and recent cannabis use (defined as past 30-day use vs former use and never use).

**Main Outcomes and Measures:**

Objective sleep was assessed using data from 7-day wrist actigraphy, and subjective sleep was assessed with the Pittsburgh Sleep Quality Index (PSQI). Primary outcomes and measures were objective sleep duration (categorized as short [<6 hours], optimal [6-8 hours], or long [>8 hours]), sleep percentage (defined as percentage of time asleep; low percentage: <85%), sleep fragmentation (defined as the index of restlessness during sleep; high fragmentation: highest quartile), and wakefulness after sleep onset (WASO; prolonged WASO: ≥30 minutes) and subjective sleep quality (PSQI score and poor sleep quality: PSQI >5).

**Results:**

A total of 1266 participants (891 with objective and 1128 with subjective sleep data) were analyzed. Among the 891 participants with objective sleep data, the mean (SD) age at the year-35 examination was 60.7 (3.6) years; the cohort included 564 females (63.3%) and 365 Black (41.0%) and 526 White (59.0%) participants. After multivariable adjustment, each additional cannabis-year was associated with higher odds of low sleep percentage (odds ratio [OR], 1.10; 95% CI, 1.06-1.14), high sleep fragmentation (OR, 1.04; 95% CI, 1.01-1.08), and prolonged WASO (OR, 1.21; 95% CI, 1.10-1.34). Compared with participants with former use and never use status, those with recent use had higher odds of low sleep percentage, high fragmentation, prolonged WASO, and long (vs optimal) sleep duration (eg, vs never use, prolonged WASO: OR, 3.23 [95% CI, 1.44-7.27]; long sleep duration: OR, 4.60 [95% CI, 1.81-11.70]). Cannabis use was not associated with subjective sleep quality.

**Conclusions and Relevance:**

In this cohort study, both cumulative use and recent cannabis use were associated with poorer objective sleep quality, although no association with self-reported sleep quality was found. These findings underscore the importance of assessing both subjective and objective sleep and suggest potential adverse association between long-term recreational cannabis exposure and midlife sleep health.

## Key Points

**Question:**

Is cumulative and recent recreational cannabis use associated with objective and subjective sleep measures in midlife adults?

**Findings:**

In this cohort study of 1266 participants (891 with objective and 1128 with subjective sleep data), both cumulative use and recent use of cannabis were associated with poorer objective sleep quality, including lower percentage, greater fragmentation, and longer wake after sleep onset; recent use was also associated with longer sleep duration. No associations were observed with subjective sleep quality.

**Meaning:**

The findings of this study suggest that recreational cannabis use may adversely affect objective sleep quality in midlife, underscoring the importance of objective sleep assessment when evaluating cannabis-related sleep outcomes.

## Teaser

This cohort study assesses the association of cumulative recreational cannabis exposure from young adulthood to middle age and recent cannabis use with objective and subjective sleep outcomes in middle age.

## Introduction

Cannabis refers to the dried leaves, flowers, stems, and seeds from the plant *Cannabis sativa* and cannabis plant–derived products.^1,2^ Over the years, cannabis use has steadily increased in the US across age groups, including as a sleep aid.^3,4,5^ This upward pattern parallels expanding medical and nonmedical cannabis legalization and declining perceptions of cannabis-related health risks.^6,7,8,9,10^

At the same time, sleep is increasingly recognized as a fundamental component of overall health, affecting cognitive performance, emotional well-being, mental health, and cardiometabolic outcomes.^11,12,13,14^ National data indicate that sleep problems are common in the US. Between 2017 and 2020, approximately 30% of adults reported difficulty sleeping,^15^ and an estimated 30% of the adult population have chronic insomnia or insomnia symptoms.^16^ Given the high prevalence of both cannabis use and sleep disturbances, understanding the association between cannabis use and sleep health has important public health implications.

Sleep health—including duration, efficiency, and quality—has demonstrated bidirectional associations with use of substances, including cannabis.^17,18^ However, evidence regarding the cannabis and sleep association remains limited and inconsistent.^19^ Recent systematic reviews and meta-analyses suggest that the use of cannabis for medical purposes may improve subjectively measured sleep but not objectively measured sleep; these findings were largely derived from small, clinic-based, short-term trials.^20,21^ Studies on recreational cannabis are also limited by cross-sectional designs^22^ and reliance on self-reported sleep measures.^23,24,25,26^ Moreover, concerns regarding long-term sleep outcomes of cannabis use have emerged,^23,27^ but longitudinal evidence remains scarce.^17,23^

Using longitudinal data from the Coronary Artery Risk Development in Young Adults (CARDIA) study with 35 years of follow-up, we conducted a study to examine the associations of cumulative cannabis exposure from young adulthood to middle age and recent cannabis use with objective and subjective sleep outcomes in middle age. We also examined whether these associations were modified by race and sex, cigarette smoking, alcohol consumption, and other substance use.

## Methods

### The CARDIA Core and Sleep Ancillary Studies

The CARDIA study recruited 5115 self-identified Black and White adults aged 18 to 30 years in 1985 to 1986 (year 0) across 4 US sites (Birmingham, Alabama; Chicago, Illinois; Minneapolis, Minnesota; and Oakland, California). Participants completed 9 follow-up examinations from year 0 through 2020 to 2022 (year 35).^28^ The Year-35 Sleep Ancillary Study collected both objective and subjective sleep measures.^29^ Both the CARDIA year-35 core and ancillary sleep studies were approved by the institutional review board (IRB) at each site. All participants in these studies provided written informed consent. The IRBs for the University of Alabama at Birmingham, Northwestern University Feinberg School of Medicine, University of Minnesota School of Public Health, and Kaiser Permanente Division of Research approved this cohort study. We followed the Strengthening the Reporting of Observational Studies in Epidemiology (STROBE) reporting guideline.

### Current Study and Analysis Samples

The CARDIA study was originally designed to recruit approximately equal numbers of Black and White young adults to facilitate investigation of racial differences in cardiovascular risk and related health outcomes. Of the 1285 participants with valid actigraphy or Pittsburgh Sleep Quality Index (PSQI) data, 19 were excluded due to pregnancy, medical cannabis use, or insufficient cannabis use data (<6 examinations or a missing year-35 examination plus ≥1 additional examination), leaving 1266 participants. Of these participants, 891 were included in analyses of objective sleep, 1128 were included in analyses of subjective sleep, and 753 contributed to both analyses. Study design, participant flow, and analytic samples are presented in eFigure 1 in Supplement 1.

### Cannabis Use Assessment

Lifetime use and recent use of nonmedical cannabis were self-assessed at each CARDIA examination using questions on ever use, lifetime frequency, and past 30-day use. At year 35, participants were categorized by recent use (past 30-day use), former use (ever used but no past 30-day use), or never use. Annual cannabis use was estimated from reported past 30-day frequency, with missing values imputed using data from the nearest available visits. Cumulative cannabis exposure was then calculated in cannabis-years (365 days of use) by summing the annual estimates over follow-up. Details of the computation of cumulative lifetime cannabis use are provided in the eAppendix in Supplement 1.^30,31,32,33,34,35^

### Objective Sleep Assessments by Wrist Actigraphy

Detailed information on the sleep data collection using wrist actigraphy has been published.^29^ Briefly, participants wore an actigraphy device (Actiwatch Spectrum PLUS; Philips Respironics) on the nondominant wrist for 7 days and completed a daily sleep log. A 7-day actigraphy protocol has been shown to provide reliable estimates of mean sleep parameters in healthy adults.^36^ Primary measures included the following: (1) sleep duration, defined as the total time (in hours) spent asleep during the primary rest interval; (2) sleep percentage, defined as the percentage of time spent asleep relative to the sleep period (the interval between sleep onset and sleep end), with higher percentages indicating better sleep quality; (3) sleep fragmentation, defined as the index of restlessness during the sleep period expressed as a percentage, with higher percentages indicating greater sleep disruption; and (4) wakefulness after sleep onset (WASO), which is measured in minutes and includes all time spent awake from the initial onset of sleep until the end of the sleep period, with higher values indicating greater sleep disruption.

Common cutoff points were used to define unfavorable vs optimal objective sleep measures. The definitions for short, optimal, and long sleep durations were less than 6 hours, 6 to 8 hours, and more than 8 hours, respectively. Low sleep percentage was less than 85%; high sleep fragmentation was within the highest quartile, and prolonged WASO was 30 or more minutes.^37^

### Subjective Sleep Quality

The PSQI assesses sleep quality across 7 domains, yielding scores from 0 to 21, with higher scores indicating poorer sleep quality. Scores were analyzed as a continuous measure and dichotomized, with scores higher than 5 indicating poor sleep quality.^38^

### Covariate Assessments

Covariates were selected a priori based on their hypothesized association with sleep and/or cannabis use. Most covariate data were collected at the CARDIA year-35 core examination and Year-35 Sleep Ancillary Study, except for cumulative lifetime cigarette and alcohol exposure and highest educational level, which were derived from repeated assessments.^29,39,40^ Sociodemographic factors included age, sex (male or female), race (Black or White), study sites, and educational level. Lifestyle factors included smoking status, alcohol consumption, physical activity, and recent use of any other substances (cocaine, crack, amphetamines, methamphetamines, or heroin). Comorbidity status included any chronic diseases (heart disease, stroke, hypertension, hypercholesterolemia, chronic obstructive pulmonary disease, and cancer). Data were collected via self-report, with medication use verified by participants. Alcohol intake (mL per day) was calculated from beer, wine, and liquor consumed per week.^41^ Physical activity was assessed using the CARDIA Physical Activity History Questionnaire and summarized as exercise units.^42,43^ Body mass index was calculated as weight in kilograms divided by height in meters squared. Depression was measured using the Center for Epidemiological Studies–Depression (CES-D) scale^44,45^ (score ranging from 0 to 60, with higher scores indicating greater distress).

Given that cigarette smoking is associated with poorer sleep outcomes^46,47^ and directly associated with cannabis use,^48^ cumulative lifetime cigarette exposure was calculated in pack-years (1 pack-year = 1 pack per day for 1 year).^32,33^ Similarly, cumulative lifetime alcohol use was estimated in drink-years (1 drink-year = 1 drink per day for 1 year).^30,31,49^

### Statistical Analysis

Analyses were performed between January 15 and July 15, 2026, using SAS, version 9.4 and SAS/STAT 15.3 (SAS Institute Inc). A 2-tailed *P* < .05 was considered statistically significant.

Participant characteristics were summarized using descriptive statistics and compared across cumulative cannabis exposure levels using χ^2^, *F* test, or Kruskal-Wallis test.^31^ Patterns of missing covariate data were examined: CES-D score had the highest missingness (21%), followed by lifestyle covariates (<4%). Multiple Imputation by Chained Equations (5 imputations) was used for adjusted analyses, and estimates were pooled using PROC MIANALYZE (SAS Institute Inc).^50^ Multivariable robust regression models were used for continuous sleep outcomes to account for nonnormality and heteroscedasticity.^51^ Logistic regression was conducted for categorical outcomes.

In the primary analyses, cumulative lifetime cannabis use through year 35 was modeled continuously. Model 1 was adjusted for sociodemographic characteristics (age, sex, race, and study sites). Model 2 was additionally adjusted for highest educational level, lifestyle factors (lifetime cigarette pack-years, lifetime alcohol drink-years, year-35 body mass index, physical activity, and other substance use), comorbidity status (any vs none), depressive symptoms, and sleep medication use. In secondary analyses, recent cannabis use status (recent, former, and never) was modeled categorically with all year-35 covariates and highest educational level.

Effect modification by race, sex, smoking, alcohol use, and other substance use was evaluated using interaction terms. In sensitivity analyses, we (1) excluded participants with recent cannabis use to determine whether history of earlier (but not recent) cannabis use among those with former use was independently associated with sleep health outcomes, (2) modeled cumulative lifetime cannabis use with restricted cubic spline (3 percentile-based knots) to assess nonlinear associations, and (3) repeated analyses among complete-case samples. Additional stratified analyses by smoking status were conducted to evaluate the associations between cannabis and sleep, independent of cigarette use.

## Results

### Sample Characteristics

A total of 891 participants with objective sleep data were included. Participants had a mean (SD) age at the year-35 examination of 60.7 (3.6) years and included 564 females (63.3%) and 327 males (36.7%), of whom 365 (41.0%) self-identified as Black and 526 (59.0%) as White (Table 1). Among 891 participants, 514 (57.7%) reported 0 (never use; n = 141) or very low (>0 to 0.5 cannabis-years; n = 373) lifetime cannabis exposure. Among 108 participants with current cannabis use, 92 (85.2%) were in the highest lifetime exposure category (>2 to 35.8 cannabis-years). Participants with higher cumulative cannabis exposure (eg, >2 to 35.8 cannabis-years) had higher prevalence of cigarette smoking (102 [56.7%]; *P* <.001), other substance use (16 [8.9%]; *P* = .03), and alcohol consumption (12.2 [0.0-27.5] mL per day; *P* < .001), compared with less exposure. Characteristics were similar between objective and subjective sleep analytic samples (eTable 1 in Supplement 1) and by recent cannabis use status (eTables 2 and 3 in Supplement 1).

**Table 1.** Sample Characteristics for Objective Sleep Outcomes, Overall and by Cumulative Lifetime Cannabis-Year Use Status

Characteristic^a^	Participants, No. (%)					*P* value^c^
	Overall (n = 891)	Never use, 0 cannabis-years cumulative lifetime exposure status (n = 141)	Ever use, cumulative lifetime exposure status, cannabis-year^b^			
			>0 to 0.5 y (n = 373)	0.5 to 2 y (n = 197)	>2 to 35.8 y (n = 180)	
Current cannabis-use status						NA
Current use	108 (12.1)	0 (0)	7 (1.9)	9 (4.6)	92 (51.1)	
Former use	642 (72.1)	0 (0)	366 (98.1)	188 (95.4)	88 (48.9)	
Never use	141 (15.8)	141 (100.0)	0 (0)	0 (0)	0 (0)	
Age, mean (SD), y	60.7 (3.6)	59.7 (3.7)	60.5 (3.6)	61.3 (3.1)	60.9 (3.6)	.005
Sex						
Female	564 (63.3)	93 (66.0)	283 (75.9)	101 (51.3)	87 (48.3)	<.001
Male	327 (36.7)	48 (34.0)	90 (24.1)	96 (48.7)	93 (51.7)	
Race						
Black	365 (41.0)	61 (43.3)	148 (39.7)	67 (34.0)	89 (49.4)	.02
White	526 (59.0)	80 (56.7)	225 (60.3)	130 (66.0)	91 (50.6)	
Study site						
Birmingham, Alabama	170 (19.1)	52 (36.9)	67 (18.0)	34 (17.3)	17 (9.4)	<.001
Chicago, Illinois	282 (31.6)	49 (34.8)	116 (31.1)	66 (33.5)	51 (28.3)	
Minneapolis, Minnesota	266 (29.9)	28 (19.9)	114 (30.6)	58 (29.4)	66 (36.7)	
Oakland, California	173 (19.4)	12 (8.5)	76 (20.4)	39 (19.8)	46 (25.6)	
Highest educational level, mean (SD), y	16.3 (2.5)	16.7 (2.5)	16.6 (2.5)	16.2 (2.3)	15.3 (2.5)	<.001
Ever cigarette smokers	298 (33.5)	7 (5.0)	92 (24.7)	97 (49.2)	102 (56.7)	<.001
Lifetime cigarette pack-years, median (IQR)^d^	0.0 (0.0-6.3)	0.0 (0.0-0.0)	0.0 (0.0-2.4)	1.6 (0.0-10.5)	3.5 (0.0-17.2)	<.001
Total exercise intensity score, mean (SD), exercise units (n = 855)	372.5 (293.6)	315.5 (244.1)	370.4 (294.7)	380.4 (276.4)	412.6 (337.4)	.04
Alcohol consumption, median (IQR), mL/d (n = 858)	2.7 (0.0-15.2)	0.0 (0.0-7.3)	2.4 (0.0-12.1)	4.8 (0.0-19.3)	12.2 (0.0-27.5)	<.001
Lifetime alcohol drink-years, median (IQR) (n = 858)^e^	11.0 (2.1-33.2)	1.4 (0.0-9.5)	8.4 (1.5-23.0)	15.9 (4.3-41.8)	33.2 (12.8-57.1)	<.001
Current drinkers (n = 858)	485 (56.5)	50 (37.0)	202 (56.0)	114 (60.0)	119 (69.2)	<.001
BMI, mean (SD) (n = 863)	30.3 (7.2)	31.1 (7.3)	30.1 (7.5)	30.6 (7.3)	29.8 (6.1)	.37
Depression or CES-D scale score, mean (SD) (n = 707)	8.9 (7.5)	8.2 (7.2)	9.0 (7.5)	8.3 (6.3)	10.2 (8.6)	.10
Use of other substances^f^	62 (7.0)	2 (1.4)	22 (5.9)	22 (11.2)	16 (8.9)	.03
Use of sleep medicine	56 (6.3)	8 (5.7)	25 (6.7)	15 (7.6)	8 (4.4)	.61
Any chronic diseases^g^	615 (69.0)	87 (61.7)	264 (70.8)	136 (69.0)	128 (71.1)	.22
Sleep duration, mean (SD), h^h^	6.8 (1.1)	6.8 (0.8)	6.8 (1.0)	6.9 (1.1)	6.8 (1.2)	.68
Sleep duration category						
Short: <6 h	185 (20.8)	24 (17.0)	78 (20.9)	39 (19.8)	44 (24.4)	.08
Optimal: 6-8 h	607 (68.1)	107 (75.9)	259 (69.4)	127 (64.5)	114 (63.3)	
Long: >8 h	99 (11.1)	10 (7.1)	36 (9.7)	31 (15.7)	22 (12.2)	
Sleep percentage, mean (SD), %^h^	90.0 (4.6)	91.2 (3.6)	90.6 (4.6)	90.1 (4.4)	87.7 (4.9)	<.001
Low sleep percentage: <85%	109 (12.2)	8 (5.7)	31 (8.3)	21 (10.7)	49 (27.2)	<.001
Sleep fragmentation, mean (SD), %^h^	21.2 (7.8)	19.8 (7.1)	20.1 (7.4)	21.3 (7.7)	24.7 (8.4)	<.001
High fragmentation (highest quartile)	222 (24.9)	21 (14.9)	78 (20.9)	54 (27.4)	69 (38.3)	<.001
WASO, mean (SD), min^h^	45.3 (22.3)	39.3 (16.6)	41.9 (21.6)	45.0 (21.5)	57.3 (24.3)	<.001
Prolonged WASO: ≥30 min	669 (75.1)	96 (68.1)	259 (69.4)	149 (75.6)	165 (91.7)	<.001

Abbreviations: BMI, body mass index (calculated as weight in kilograms divided by height in meters squared); CES-D, Center for Epidemiological Studies–Depression scale; NA, not applicable; WASO, wakefulness after sleep onset.

^a^ All characteristics were collected at the CARDIA year-35 core examination and Year-35 Sleep Ancillary Study, except for highest educational level and cumulative lifetime cigarette and alcohol exposure, which were derived from data collected across all prior examination years.

^b^ In cannabis-years, 1 cannabis-year of exposure is equivalent to 365 days of cannabis use.

^c^ *P* values for group comparisons were based on χ^2^ tests, F tests, or Kruskal-Wallis tests.

^d^ In pack-years, 1 pack-year of exposure is equivalent to 7300 cigarettes (1 y × 365 d/y × 1 pack/d × 20 cigarettes/pack).

^e^ In drink-years, 1 drink-year is the total amount of ethanol consumed by a person who had 1 alcoholic drink per day for 1 year (1 drink-year = 17.24 mL of ethanol/drink × 1 drink/d × 365 d/y = 6292.6 mL of ethanol).

^f^ Any use of cocaine, crack, amphetamines, methamphetamines, or heroin.

^g^ Any chronic diseases, including heart disease, stroke, hypertension, hypercholesterolemia, chronic obstructive pulmonary disease, and cancer.

^h^ Assessed using a 7-day wrist actigraphy device.

Objectively measured sleep showed a mean (SD) duration of 6.8 (1.1) hours, 90% (4.6%) sleep percentage, 21% (7.8%) sleep fragmentation, and 45.3 (22.3) minutes WASO. Mean (SD) PSQI score was 6.3 (3.8), with 566 participants (50.2%) scoring higher than 5.

### Primary Associations With Lifetime Cannabis Use

Table 2 summarizes the association between lifetime cannabis exposure and continuous sleep outcomes. After adjusting for age, race, sex, and study sites (model 1), each additional cannabis-year was associated with lower sleep percentage (β = –0.21%; 95% CI, –0.27% to –0.16%), higher sleep fragmentation (β = 0.26%; 95% CI, 0.15%-0.36%), and longer WASO (β = 1.05 minutes; 95% CI, 0.78-1.32 minutes), but not associated with sleep duration or PSQI scores. Associations remained significant after further adjustment (model 2). Excluding participants with recent cannabis use yielded similar results (Table 2), as did models that substituted current cigarette smoking and current alcohol use for their lifetime measures.

**Table 2.** Association Between Cumulative Lifetime Cannabis Use and Sleep Health^a^

Sleep characteristic	β (95% CI)^b^	
	Model 1^c^	Model 2^d^
**Main analysis sample**		
Actigraphy data (n = 891)		
Sleep duration, h	−0.001 (−0.01 to 0.01)	0.0002 (−0.01 to 0.02)
Sleep percentage, %	−0.21 (−0.27 to −0.16)	−0.18 (−0.24 to −0.12)
Sleep fragmentation, %	0.26 (0.15 to 0.36)	0.20 (0.09 to 0.31)
WASO, min	1.05 (0.78 to 1.32)	0.85 (0.57 to 1.14)
Self-reported data (n = 1128)		
Sleep quality or PSQI score	0.03 (−0.02 to 0.07)	−0.003 (−0.05 to 0.04)
**Main analysis sample excluding participants with current cannabis use**		
Actigraphy data (n = 783)		
Sleep duration, h	−0.01 (−0.02 to 0.002)	−0.01 (−0.03 to 0.02)
Sleep percentage, %	−0.27 (−0.30 to −0.23)	−0.23 (−0.32 to −0.14)
Sleep fragmentation, %	0.22 (0.15 to 0.29)	0.17 (−0.0007 to 0.34)
WASO, min	1.26 (1.08 to 1.44)	1.10 (0.66 to 1.54)
Self-reported data (n = 1002)		
Sleep quality or PSQI score	0.01 (−0.01 to 0.04)	−0.02 (−0.08 to 0.04)

Abbreviations: PSQI, Pittsburgh Sleep Quality Index; WASO, wakefulness after sleep onset.

^a^ Derived from repeated Coronary Artery Risk Development in Young Adults study cannabis-use assessments and calculated as cannabis-years (1 cannabis-year = 365 days of cannabis use).

^b^ Expected change in sleep measures per each additional cannabis-year of lifetime cannabis exposure.

^c^ Model 1 was adjusted for year-35 age, sex, race, and study site.

^d^ Model 2 was additionally adjusted for highest educational level, year-35 recent use of other substances, physical activity, body mass index, sleep medication use, Center for Epidemiological Studies–Depression scale score, comorbidity status, lifetime cigarette pack-years, and lifetime drink-years, with missing covariate values imputed.

For categorical sleep outcomes (Table 3), each additional cannabis-year was associated with higher odds of low sleep percentage (odds ratio [OR], 1.10; 95% CI, 1.06-1.14), high sleep fragmentation (OR, 1.04; 95% CI, 1.01-1.08), and prolonged WASO (OR, 1.21; 95% CI, 1.10-1.34). There was no association with sleep duration categories or poor subjective sleep quality.

**Table 3.** Adjusted Odds Ratios for Sleep Outcomes by Cumulative Lifetime Cannabis Use^a^^,^^b^

Sleep characteristic	AOR (95% CI)^c^
**Main analysis sample**	
Actigraphy data (n = 891)	
Sleep duration category	
Short vs optimal: 6-8 h	1.01 (0.98-1.05)
Long vs optimal: 6-8 h	1.02 (0.97-1.06)
Low sleep percentage: <85%	1.10 (1.06-1.14)
High sleep fragmentation (highest quartile)	1.04 (1.01-1.08)
Prolonged WASO: ≥30 min	1.21 (1.10-1.34)
Self-reported data (n = 1128)	
Poor sleep quality or PSQI score >5	1.01 (0.98-1.04)
**Main analysis sample excluding participants with current cannabis use**	
Actigraphy data (n = 783)	
Sleep duration category	
Short vs optimal: 6-8 h	1.04 (0.98-1.1)
Long vs optimal: 6-8 h	1.02 (0.93-1.11)
Low sleep percentage: <85%	1.19 (1.11-1.28)
High sleep fragmentation (highest quartile)	1.03 (0.98-1.09)
Prolonged WASO: ≥30 min	1.16 (1.04-1.31)
Self-reported data (n = 1002)	
Poor sleep quality or PSQI score >5	0.99 (0.95-1.04)

Abbreviations: AOR, adjusted odds ratio; PSQI, Pittsburgh Sleep Quality Index; WASO, wakefulness after sleep onset.

^a^ Derived from repeated Coronary Artery Risk Development in Young Adults study cannabis-use assessments and calculated as cannabis-years (1 cannabis-year = 365 days of cannabis use).

^b^ Model was adjusted for year-35 age, sex, race, study site, highest educational level, year-35 body mass index, physical activity, sleep medication use, Center for Epidemiological Studies–Depression scale score, comorbidity status, recent use of other substances, lifetime cigarette pack-years, and lifetime alcohol drink-years, with missing covariate values imputed.

^c^ Odds ratio for the sleep outcome associated with each additional cannabis-year of lifetime cannabis exposure.

### Restricted Cubic Spline Analyses

The overall findings from the restricted cubic splines were consistent with the primary models (eFigure 2 in Supplement 1). There was no evidence of nonlinearity for sleep percentage, sleep fragmentation, and PSQI scores (all *P *> .05 for spline components). Modest curvature was observed for sleep duration and WASO, primarily at low levels of cannabis exposure, but linear models provided adequate fit.

### Recent Cannabis Use

Associations between recent cannabis use at year 35 and continuous sleep outcomes are shown in Figure 1. Compared with participants with never use status, those with recent use had longer sleep duration (β = 0.33 hours; 95% CI, 0.07-0.59 hours), lower sleep percentage (β = −1.74%; 95% CI, −2.78% to −0.70%), higher sleep fragmentation (β = 2.14%; 95% CI, 0.16%-4.13%), and longer WASO (β = 11.09 minutes; 95% CI, 6.03-16.16 minutes). Similar patterns were observed when comparing participants with recent use vs former use. Those with former use did not differ from those with never use status on any objective sleep measure.

**Figure 1.**
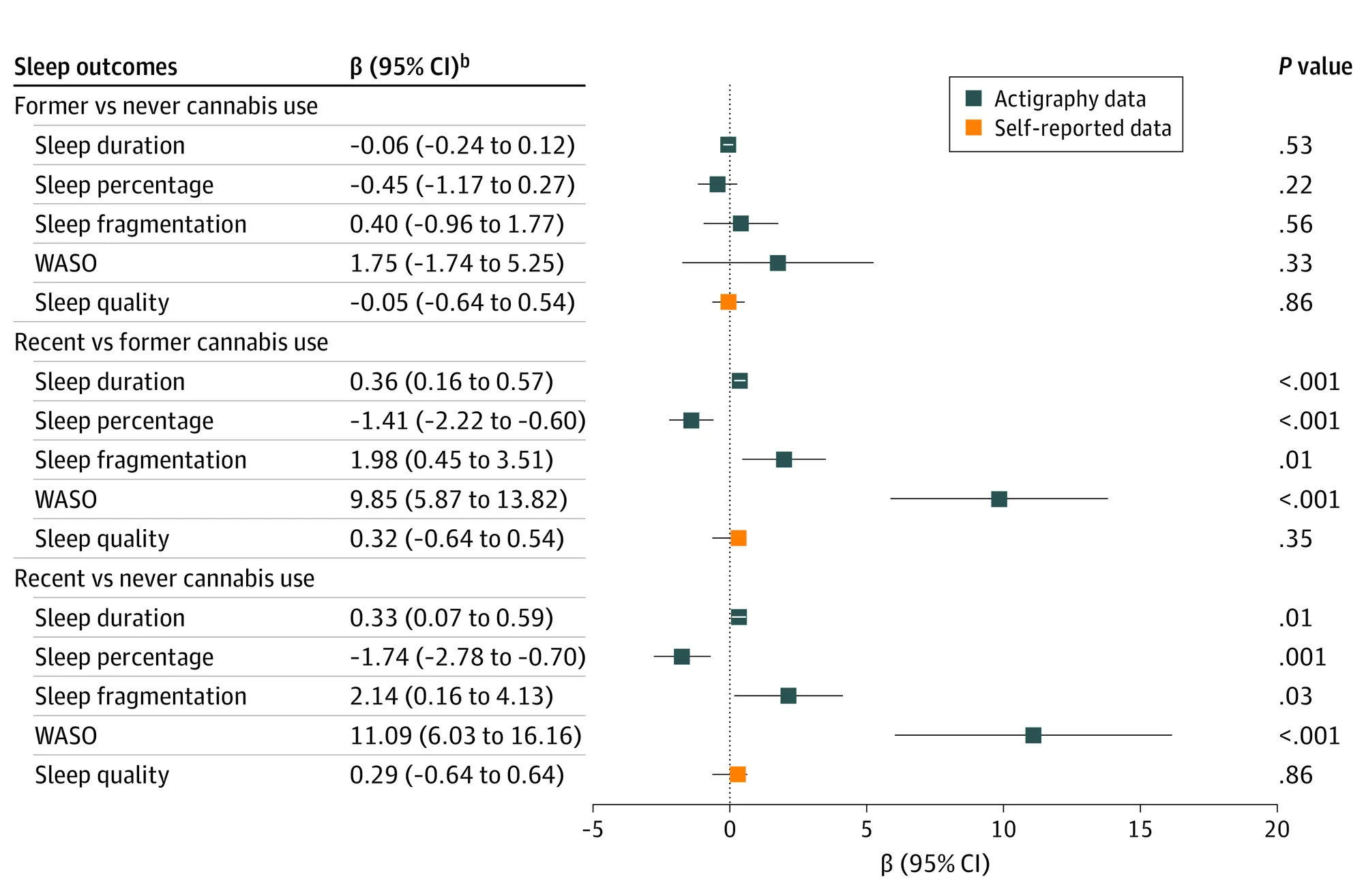
Dot Plot of Association Between Year-35 Recent Cannabis Use and Sleep Health^a^ WASO indicates wakefulness after sleep onset. Error bars represent 95% CIs. ^a^Model was adjusted for year-35 age, sex, race, study site, highest educational level, cigarette smoking status, alcohol drinking status, recent use of other substances, physical activity, body mass index, sleep medication use, Center for Epidemiological Studies–Depression scale score, and comorbidity status, with missing covariate values imputed. ^b^Expected difference in sleep measures between pairs of cannabis use categories (recent, former, and never).

For categorical sleep outcomes (Figure 2), participants with recent use had higher odds of low sleep percentage, high fragmentation, prolonged WASO, and long (vs optimal) sleep duration (eg, vs never use: prolonged WASO: OR, 3.23 [95% CI, 1.44-7.27]; long sleep duration: OR, 4.60 [95% CI, 1.81-11.70]). No association was found with scores or dichotomized PSQI sleep quality.

**Figure 2.**
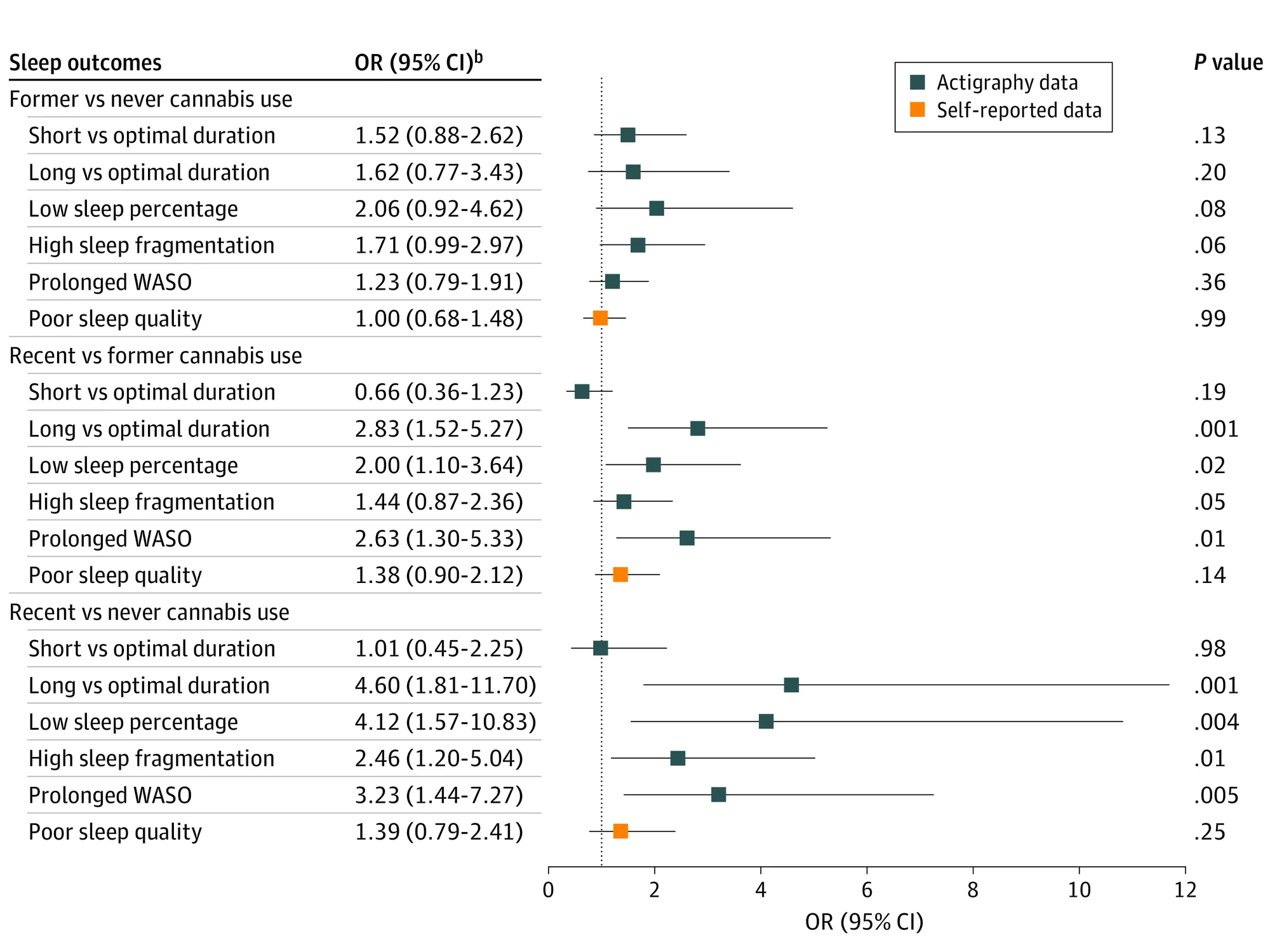
Dot Plot of Adjusted Odds Ratios (ORs) for Sleep Health Outcomes Associated With Recent Cannabis Use Status^a^ WASO indicates wakefulness after sleep onset. Error bars represent 95% CIs. ^a^Model was adjusted for year-35 age, sex, race, study site, highest educational level, cigarette smoking status, alcohol drinking status, recent use of other substances, physical activity, body mass index, sleep medication use, Center for Epidemiological Studies–Depression scale score, and comorbidity status, with missing covariate values imputed. ^b^OR for the sleep outcome between pairs of cannabis use categories (recent, former, and never).

### Additional Sensitivity and Stratification Analyses

Complete-case and stratified analyses by smoking status produced results consistent with the primary findings (eTables 4 and 5 in Supplement 1). No significant interactions were observed by race or smoking status, whereas significant interactions were detected by sex, alcohol use, and other substance use. Associations were of greater magnitude in females and noncurrent drinkers and persisted among participants without recent other substance use (eTables 6-8 in Supplement 1). For example, each additional cannabis-year was associated with higher sleep fragmentation among noncurrent drinkers (β = 0.36%; 95% CI, 0.15%-0.56%), whereas the effect size was lower among current drinkers (β = 0.18%; 95% CI, 0.04%-0.32%) (eTable 7 in Supplement 1). Stratified analyses among participants with recent other substance use were not performed because of the small sample size (n = 62).

## Discussion

In this large, community-based cohort with more than 3 decades of follow-up, higher cumulative cannabis use was associated with poorer objective measures of sleep quality, including lower efficiency and greater disruptions in middle age, independent of sociodemographic factors, health behaviors, and comorbidities. These associations remained evident when sleep measures were categorized using commonly applied thresholds, with higher cumulative cannabis exposure associated with greater odds of unfavorable sleep outcomes. Recent cannabis use was also associated with poorer objective sleep quality and longer (vs optimal) sleep duration. In contrast, neither lifetime nor recent cannabis use was associated with subjective sleep quality. Findings were generally consistent across race, sex, and smoking status, with slightly larger effect sizes observed among females and noncurrent drinkers.

The discrepancy between objective and subjective sleep findings may have important clinical and public health implications, particularly given that cannabis is increasingly perceived and promoted as a sleep aid.^3,4,5,52^ Individuals may not perceive declines in sleep quality or may interpret longer sleep duration as improved sleep despite objectively poorer sleep. Thus, cannabis-related sleep disturbances may go unrecognized. Public health messaging, clinical awareness, and counseling should emphasize that perceived sleep benefits may not correspond to objective sleep health and that longer sleep duration may not necessarily represent an optimal sleep pattern.

Our findings extend the growing but methodologically limited literature on recreational cannabis use and objectively measured sleep.^22,25,27,53,54,55,56,57^ For example, actigraphy-based research in 178 young adults found that greater daily cannabis use was associated with lower sleep efficiency and more awakenings.^54^ Similarly, a cross-sectional polysomnography study of 177 healthy adults found that both acute use within 3 hours of sleep and frequent use (>20 days per month) were associated with greater WASO and stage 1 (light) sleep.^22^ Our results align most closely with the findings by Velzeboer et al,^27^ who reported shorter sleep duration and poorer sleep quality among participants with long-term daily use in a large polysomnography-based sample of individuals with obstructive sleep apnea (n = 1449). In contrast, a large population-based cross-sectional study from Brazil, which included both subjective and objective (polysomnography) sleep measures, reported no association between sleep and cannabis use; however, it modeled sleep parameters as factors in cannabis use, limiting comparability with our study.^58^

To date, objective sleep data among individuals using recreational cannabis remain scarce. Although the available evidence generally suggests poorer objective sleep quality among individuals who use recreational cannabis, most studies are limited by small samples, cross-sectional designs, restricted exposure assessments, clinical samples, or short follow-up periods.^57^ The literature on the association between recreational cannabis use and subjective sleep outcomes provides mixed results. Some population-based studies report poorer self-reported sleep among individuals with recent and/or heavy cannabis use,^24,25,26,59,60^ whereas other research describes short-term improvement.^23,52,61,62^ Methodological differences likely contribute to this heterogeneity. Many prior studies were cross-sectional, focused on younger populations or those exclusively using cannabis, or assessed single sleep dimensions such as duration, rather than multidimensional measures. In our cohort, we observed no association between cannabis use and global PSQI scores. In our study, the older age of participants and the high proportion of individuals with long-term and heavier use among those with recent use (85.2%) may have attenuated short-term associations observed in younger populations.^61,62^

Our results differ from parts of the medical cannabis literature, where some meta-analyses report small improvements in subjective sleep quality.^20,63^ However, these studies are typically short-term trials (≤8 weeks), clinic-based, and/or assess sleep as a secondary outcome. Reviews of objective sleep outcomes with medical cannabis also report inconsistent or null findings, with much of the evidence considered to be low certainty.^21^ Our findings highlight the importance of considering long-term cannabis exposure in chronic sleep conditions, as potential long-term associations may not be captured in short-term studies.

One possible explanation for the small improvement in subjective sleep quality observed in these medical cannabis studies is that most participants were from clinical populations with pain, cancer, or sleep disorders. Cannabis may improve perceived sleep quality by reducing symptoms that interfere with sleep, even when objective sleep measures show little or no change.

Tetrahydrocannabinol (THC) and cannabidiol (CBD), the major cannabinoids in cannabis,^64^ may affect sleep through distinct endocannabinoid pathways.^65^ THC may reduce sleep latency but may also disrupt normal sleep architecture, increase fragmentation, and lead to tolerance over time, whereas CBD is promoted as a sleep aid because of its potential anxiolytic and analgesic properties.^66^ Heterogeneity across studies may also be explained by differences in cannabinoid composition, dose, and route of administration.^26,67,68^ Nonetheless, evidence on CBD and objective sleep outcomes remains inconsistent, and most randomized trials have had short follow-up periods, which are insufficient for evaluating long-term outcomes.^20^

### Strengths and Limitations

This study has several key strengths. Its prospective design with repeated cannabis assessments for more than 35 years allowed us to capture both recent and cumulative exposures.^31,33^ We also leveraged both objective and subjective sleep measures and included a large, racially diverse, population-based cohort. Moreover, the observed associations were independent of cigarette smoking, alcohol use, and other substance use, supporting an independent cannabis-specific association with sleep outcomes. This finding is particularly relevant in settings where cannabis and cigarette use commonly co-occur,^69,70^ complicating the attribution of substance-specific effects.

Several limitations warrant consideration. Cannabis use was self-reported and lacked detailed information on product potency, mode of administration, and timing relative to sleep. Because cannabis potency, availability, and legal status have changed substantially over 35 years, cumulative cannabis exposure may not fully reflect biologically active exposure across calendar time. Although cumulative exposure reflects overall use, it does not characterize the timing, duration, or patterns of use, and participants with similar cumulative exposure may have had different use trajectories. While we examined recent cannabis use separately and conducted sensitivity analyses excluding those with recent use, future studies with detailed longitudinal characterization of cannabis exposure are needed. Objective sleep was measured only using a single 7-day period at the year-35 examination; therefore, changes in sleep patterns over time and the temporal relationship between cannabis exposure and sleep outcomes could not be established. Residual confounding remains possible despite extensive adjustment for lifestyle and clinical factors. Reduced participation during the COVID-19 pandemic, particularly for in-person assessments, may also have introduced selection bias. Finally, findings from this midlife US cohort may not generalize to younger populations or to higher-potency contemporary cannabis products.

## Conclusions

In this cohort study of middle-aged adults, cumulative lifetime cannabis use was associated with poorer objective sleep quality, and recent use was associated with poorer objective sleep quality and longer sleep duration, with no associations observed for subjective sleep. These findings underscore the importance of incorporating both perceived and physiologic sleep outcomes and considering the long-term association between cannabis and sleep. Future longitudinal, mechanistic, and randomized studies with long-term follow-up and objective sleep assessments are needed to clarify how cannabis use relates to sleep over time.

## Notes

Supplement 1. **eFigure 1.** Flowchart of Study Design and Analytic Samples **eFigure 2.** Predicted Sleep Outcomes by Cumulative Lifetime Cannabis Use From Restricted Cubic Splines (RCS) Models **eTable 1.** Analysis Sample Characteristics for Subjective Sleep Outcomes, Overall and by Cumulative Lifetime Cannabis-Year Use Status **eTable 2.** Analysis Sample Characteristics for Objective Sleep Outcomes, Overall and by Cumulative Lifetime Cannabis-Year Use Status **eTable 3.** Analysis Sample Characteristics for Subjective Sleep Outcomes by Recent Cannabis Use Status **eTable 4.** Association of Cumulative Lifetime Cannabis Use With Sleep Health Outcomes (Complete-Case Analyses) **eTable 5.** Association of Cumulative Lifetime Cannabis Use With Sleep Health by Smoking Status **eTable 6.** Association of Cumulative Lifetime Cannabis Use With Sleep Health by Sex **eTable 7.** Association of Cumulative Lifetime Cannabis Use With Sleep Health by Drinking Status **eTable 8.** Association of Cumulative Lifetime Cannabis Use With Sleep Health by Recent Use of Other Substances **eAppendix.** Missing Data Imputation and Computation of Cumulative Lifetime Cannabis Use
eReferences

Supplement 2.
Data Sharing Statement
