# Supplementary material for: Recent and Cumulative Cannabis Use and Sleep in Middle-Aged Adults
Source: JAMA Netw Open. 2026 Oct 9;9(10):e2638303. doi: 10.1001/jamanetworkopen.2026.38303 (PMC13653285; doi:10.1001/jamanetworkopen.2026.38303)
Supplement: Supplementary file 1 [file jamanetwopen-e2638303-s001.pdf]

Supplemental Online Content

Vu THT, Knutson KL, Auer R, Jakob J, Lloyd-Jones DM, Carnethon MR. Recent and cumulative cannabis use and sleep in middle-aged adults. *JAMA Netw Open*. 2026;9(10):e2638303. doi:10.1001/jamanetworkopen.2026.38303

eFigure 1. Flowchart of Study Design and Analytic Samples

eFigure 2. Predicted Sleep Outcomes by Cumulative Lifetime Cannabis Use From Restricted Cubic Splines (RCS) Models

eTable 1. Analysis Sample Characteristics for Subjective Sleep Outcomes, Overall and by Cumulative Lifetime Cannabis-Year Use Status

eTable 2. Analysis Sample Characteristics for Objective Sleep Outcomes, Overall and by Cumulative Lifetime Cannabis-Year Use Status

eTable 3. Analysis Sample Characteristics for Subjective Sleep Outcomes by Recent Cannabis Use Status

eTable 4. Association of Cumulative Lifetime Cannabis Use With Sleep Health Outcomes (Complete-Case Analyses)

eTable 5. Association of Cumulative Lifetime Cannabis Use With Sleep Health by Smoking Status

eTable 6. Association of Cumulative Lifetime Cannabis Use With Sleep Health by Sex

eTable 7. Association of Cumulative Lifetime Cannabis Use With Sleep Health by Drinking Status

eTable 8. Association of Cumulative Lifetime Cannabis Use With Sleep Health by Recent Use of Other Substances

eAppendix. Missing Data Imputation and Computation of Cumulative Lifetime Cannabis Use

eReferences

This supplemental material has been provided by the authors to give readers additional information about their work.

© 2026 Vu THT et al. *JAMA Network Open*.

eFigure 1: Flowchart of the study design and analytic samples

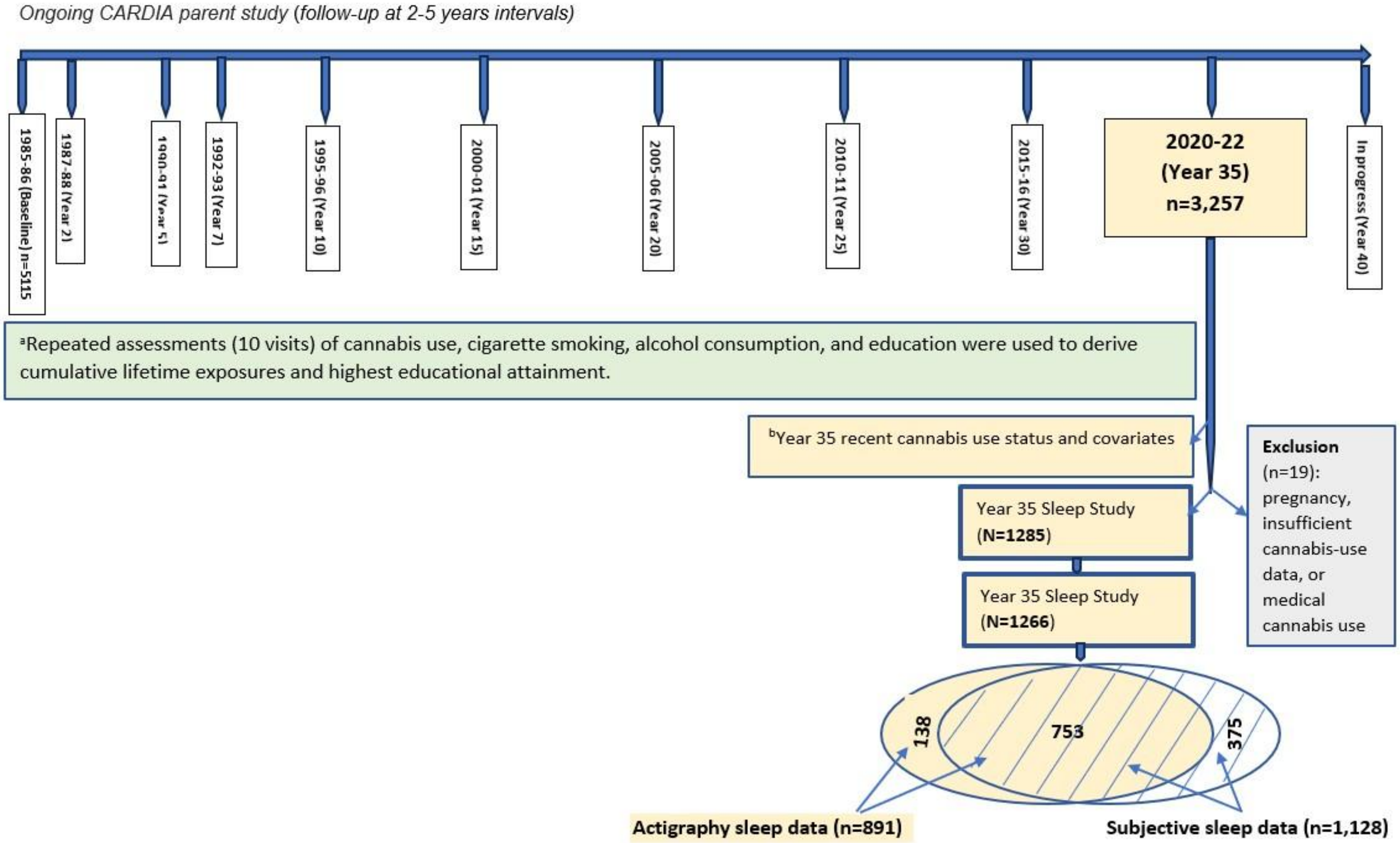

[a] Used for analyses of cumulative lifetime cannabis use and Year-35 sleep outcomes, with adjustment for other Year-35 characteristics.
[b]Used for cross-sectional analyses of Year-35 recent cannabis use and Year-35 sleep outcomes.

eFigure 2: Predicted Sleep Outcomes by Cumulative Lifetime Cannabis Use[a] from Restricted Cubic Splines (RCS) Models[b]

2A: Sleep duration (hrs)

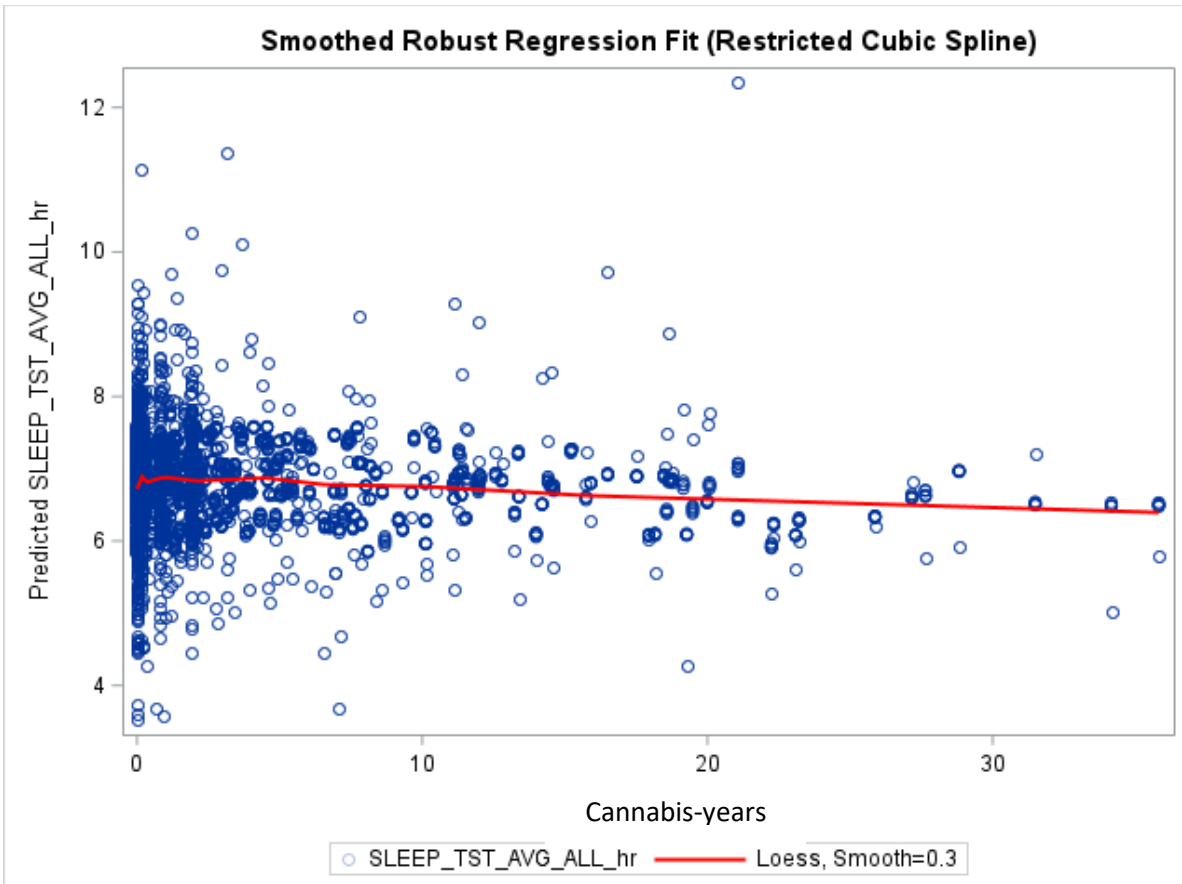

2B: Sleep percentage (%)

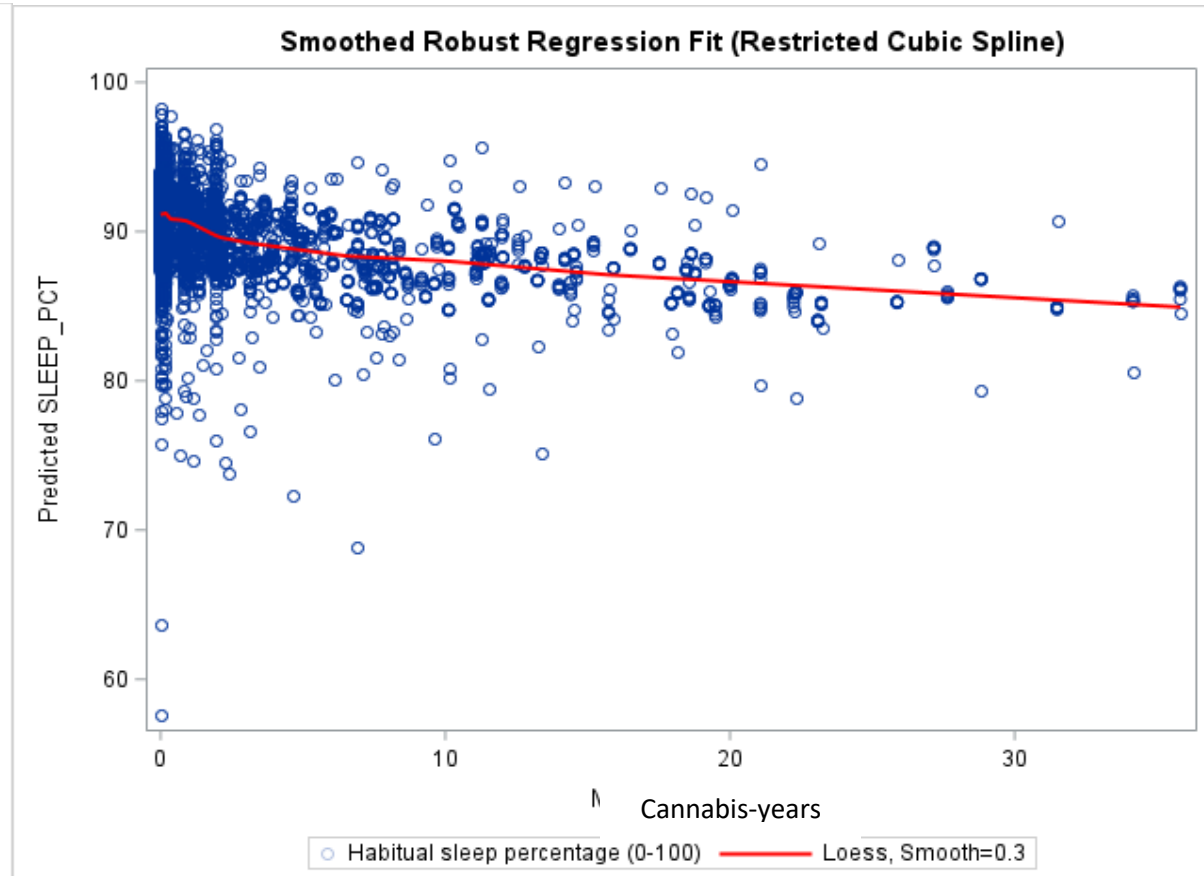

2C: Sleep Fragmentation (%)

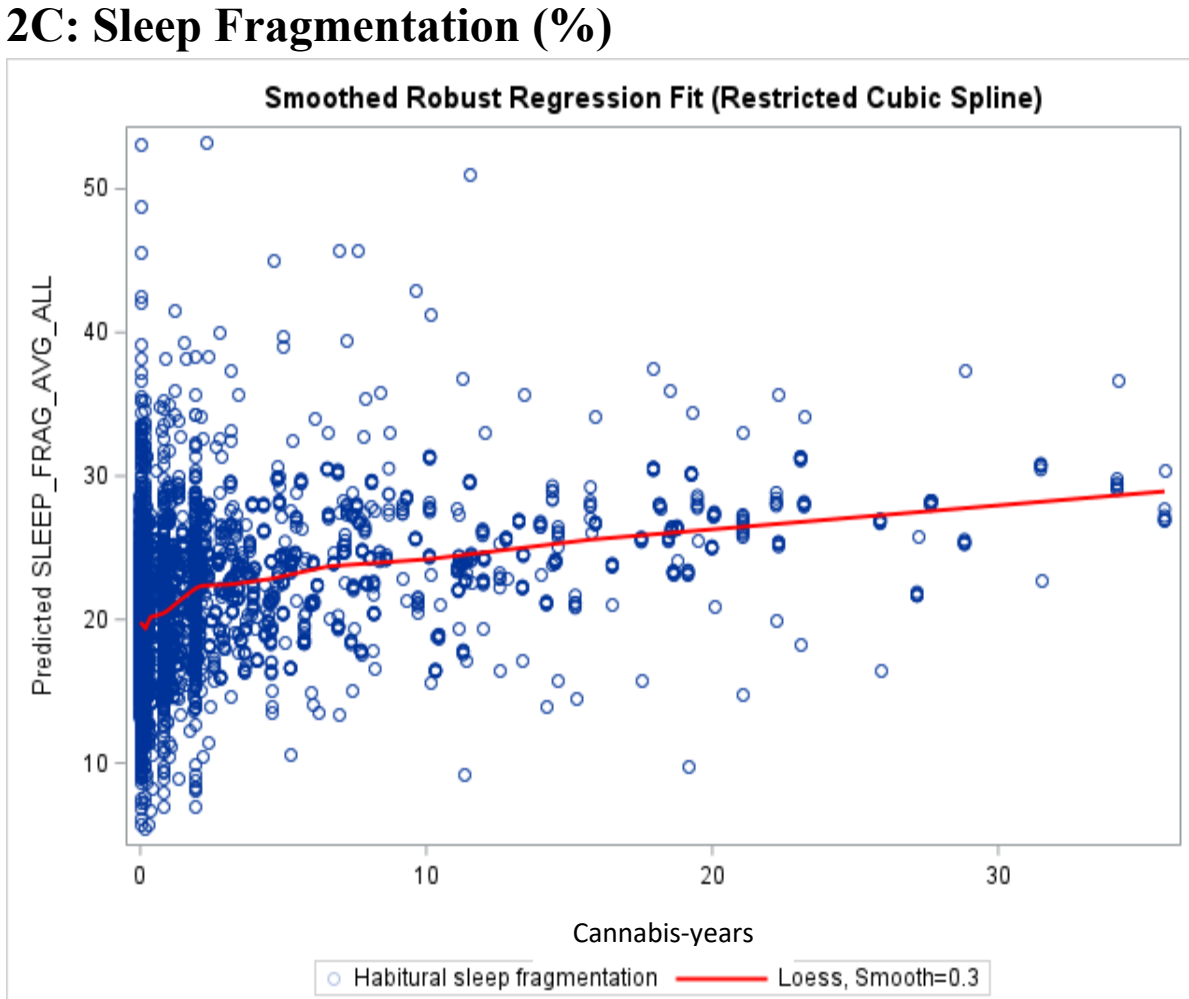

2D: Sleep WASO (Minutes)

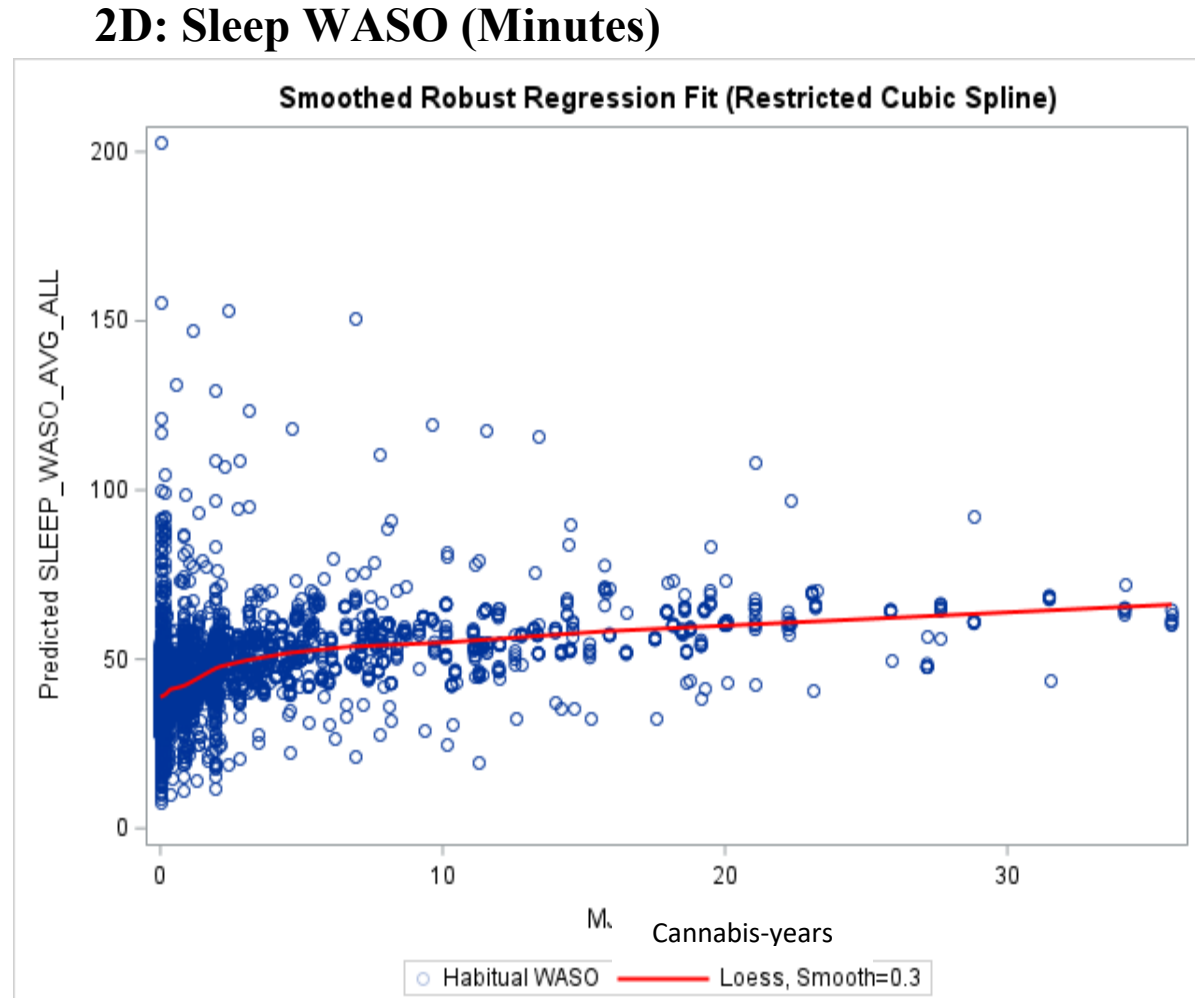

2E: Subjective Sleep Quality (PSQI score)

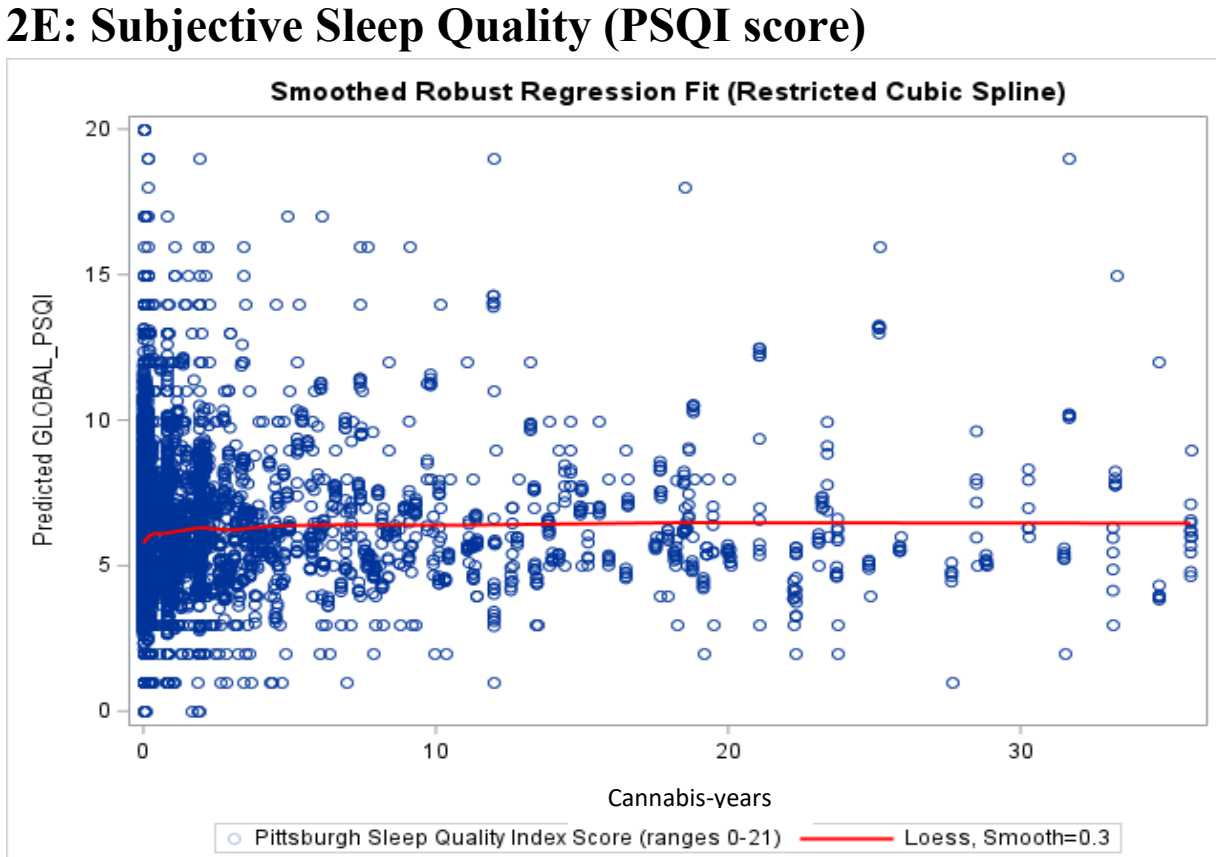

[a]Derived from repeated CARDIA cannabis-use assessments and calculated as cannabis-years (1 cannabis-year = 365 days of cannabis use).

[b]Models using restricted cubic splines with three knots were adjusted for Year-35 age, sex, race, study site, highest educational attainment, Year-35 recent use of other substances, physical activity, BMI, sleep medication use, CES-D score, comorbidity status, lifetime cigarette pack-years, and lifetime drink-years.

© 2026 Vu THT et al. *JAMA Network Open*.

eTable 1: Sample Characteristics for Subjective Sleep Outcomes, Overall and by Cumulative Lifetime Cannabis-Year Use Status

Characteristics[a]	Participants, No. (%)					P-value[c]
	Overall (n=1,128)	Cumulative lifetime exposure status				
		Never use	Ever use, cannabis-year[b]			
			> 0 to 0.5 y	0.5 to 2 y	2 to 35.8 y	
		(n=172)	(n=489)	(n=241)	(n=226)	
Current cannabis-use status						
Current use	126 (11.2)	0(0)	8 (1.6)	11 (4.6)	107 (47.4)	NA
Former use	830 (73.6)	0(0)	481 (98.4)	230 (95.4)	119 (52.7)	
Never use	172 (15.3)	172 (100.0)	0(0)	0(0)	0(0)	
Age, mean (SD), y	60.5 (3.6)	59.3 (3.9)	60.4 (3.7)	61.2 (3.1)	60.9 (3.6)	<.001
Sex						
Female	690 (61.2)	112 (65.1)	346 (70.8)	126 (52.3)	106 (46.9)	<.001
Male	438 (38.8)	60 (34.9)	143 (29.2)	115 (47.7)	120 (53.1)	
Race						
Black	463 (41.1)	77 (44.8)	199 (40.7)	85 (35.3)	102 (45.1)	.12
White	665 (59.0)	95 (55.2)	290 (59.3)	156 (64.7)	124 (54.9)	
Study Site						
Birmingham, Alabama	104 (18.1)	64 (37.2)	82 (16.8)	37 (15.4)	21 (9.3)	<.001
Chicago, Illinois	343 (30.4)	56 (32.6)	148 (30.3)	78 (32.4)	61 (27.0)	
Minneapolis, Minnesota	260 (23.1)	29 (16.9)	115 (23.5)	56 (23.2)	60 (26.6)	
Oakland, California	321 (28.5)	23 (13.4)	144 (29.5)	70 (29.1)	84 (37.2)	
Highest educational level, mean (SD), y	16.3 (2.5)	16.8 (2.6)	16.6 (2.5)	16.2 (2.4)	15.6 (2.4)	<.001
Ever cigarette smokers	383 (34.0)	5 (2.9)	120 (24.5)	125 (51.9)	133 (58.9)	<.001

© 2026 Vu THT et al. *JAMA Network Open*.

Characteristics[a]	Participants, No. (%)					P-value[c]
	Overall (n=1,128)	Cumulative lifetime exposure status				
		Never use	Ever use, cannabis-year[b]			
			> 0 to 0.5 y	0.5 to 2 y	2 to 35.8 y	
		(n=172)	(n=489)	(n=241)	(n=226)	
Lifetime pack-years of cigarette smoking [d], median (IQR)	0.0 (0.0, 5.6)	0.0 (0.0, 0.0)	0.0 (0.0, 2.0)	1.6 (0.0, 11.0)	3.7 (0.0, 16.3)	<.001
Total exercise intensity score[e], mean (SD), exercise units	372.7 (300.1)	327.8 (256.7)	375.7 (304.6)	393.3 (291.9)	377.7 (326.4)	.17
Alcohol consumption[f], mL/day, median (IQR)	2.7 (0.0, 14.7)	0.0 (0.0, 4.9)	2.4 (0.0, 12.2)	4.9 (0.0, 18.2)	9.9 (0.0, 28.5)	<.001
Lifetime alcohol drink-years[f,g], median (IQR)	11.1 (2.1, 32.7)	1.0 (0.0, 8.2)	8.6 (1.8, 23.5)	16.0 (4.2, 38.0)	31.4 (11.8, 61.5)	<.001
Current drinkers[f]	630 (56.6)	60 (35.7)	280 (57.7)	146 (61.3)	144 (64.6)	<.001
BMI, mean (SD) [h]	30.3 (7.3)	31.7 (7.8)	30.1 (7.5)	30.5 (7.1)	29.6 (6.3)	.03
Depression or CES-D scale score, mean (SD) [i]	9.3 (7.9)	7.7 (7.0)	9.2 (7.8)	9.5 (7.8)	10.6 (8.7)	.02
Use of other substances [j]	75 (6.7)	1 (0.6)	26 (5.3)	24 (10.0)	24 (10.6)	<.001
Use of sleep medicine	69 (6.1)	10 (5.8)	32 (6.5)	16 (6.6)	11 (4.9)	.82
Any chronic diseases [k]	790 (70.0)	112 (65.1)	345 (70.1)	173 (71.8)	160 (70.8)	.48
PSQI score, mean (SD)[l]	6.3 (3.8)	6.0 (3.7)	6.3 (3.7)	6.3 (3.8)	6.8 (4.1)	.19
PSQI score >5 [l]	566 (50.2)	78 (45.4)	239 (48.9)	117 (51.5)	101 (55.3)	.22

Abbreviations: BMI, body mass index (calculated as weight in kilograms divided by height in meters squared); CES-D, Center for Epidemiological Studies–Depression scale; NA, not applicable;

[a] *All characteristics were collected at the CARDIA year-35 core examination and Year-35 Sleep Ancillary Study, except for highest educational attainment and cumulative lifetime cigarette and alcohol exposure which were derived from data collected across all prior examination years.*

[b] *In cannabis-years: 1 cannabis-year of exposure is equivalent to 365 days of cannabis use.*

[c] *P-values for group comparisons based on χ^2 tests, F-tests, or Kruskal-Wallis tests.*

[d] *In pack-years: one pack-year of exposure equivalent to 7300 cigarettes (1 year x 365 days/y x 1 pack/d x 20 cigarettes/pack).*

[e] *n= 1112.*

[f] *n=1114.*

© 2026 Vu THT et al. *JAMA Network Open*.

[g] *In drink-years: one drink-year is the total amount of ethanol consumed by a person who had 1 alcoholic drink per day for 1 year (1 drink-year = 17.24 ml of ethanol/drink x 1 drink/d x 365 days/y = 6292.6 ml of ethanol).*
[h] *n= 1115*
[i] *n=836.*
[j] *Any use of cocaine, crack, amphetamines, methamphetamines, or heroin.*
[k] *Any chronic diseases including heart diseases, stroke, hypertension, hypercholesterolemia, chronic obstructive pulmonary disease, or cancer*
[l] *Pittsburgh Sleep Quality Index (PSQI) score assessed via self-reported questionnaires.*

© 2026 Vu THT et al. *JAMA Network Open*.

eTable 2: Sample Characteristics for Objective Sleep Outcomes by Recent Cannabis Use Status

Characteristics[a]	Participants, No. (%)			P-value [b]
	Year-35 Recent Cannabis Use Status			
	Current use (n=108)	Former use (n=642)	Never use (n=141)	
Age, mean (SD), y	60.5 (3.6)	60.9 (3.5)	59.7 (3.7)	.002
Sex				
Male	54 (50.0%)	225 (35.0%)	48 (34.0%)	.009
Female	54 (50.0%)	417 (65.0%)	93 (66.0%)	
Race				
White	60 (55.6%)	386 (60.1%)	80 (56.7%)	.56
Black	48 (44.4%)	256 (39.9%)	61 (43.3%)	
Study Site				
Birmingham, Alabama	9 (8.3%)	109 (17.0%)	52 (36.9%)	<.001
Chicago, Illinois	28 (25.9%)	205 (31.9%)	49 (34.8%)	
Minneapolis, Minnesota	49 (45.4%)	189 (29.4%)	28 (19.9%)	
Oakland, California	22 (20.4%)	139 (21.7%)	12 (8.5%)	
Highest educational level, mean (SD), y[c]	15.8 (2.7)	16.2 (2.5)	16.7 (2.5)	.02
Ever cigarette smokers	59 (54.6%)	232 (36.1%)	7 (5.0%)	<.001
Total exercise intensity score[d], mean (SD), exercise units	434.6 (338.1)	374.4 (293.5)	315.5 (244.1)	.008
Alcohol consumption[e], mL/day, median (IQR)	12.6 (2.6, 30.6)	2.7 (0.0, 14.8)	0.0 (0.0, 7.3)	<.001
Current alcohol drinkers[e]	79 (**76.0%**)	356 (**57.5%**)	50 (**37.0%**)	<.001
BMI, mean (SD)[f]	30.0 (5.7)	30.2 (7.4)	31.1 (7.3)	.38
Depression or CES-D scale score, mean (SD)[g]	9.7 (7.4)	8.9 (7.5)	8.2 (7.2)	.34
Use of other substances[h]	23 (21.3%)	37 (5.8%)	2 (1.4%)	<.001
Use of sleep medicine	11 (10.2%)	37 (5.8%)	8 (5.7%)	.20
Any chronic diseases[i]	81 (75.0%)	447 (69.6%)	87 (61.7%)	.07
Sleep duration, mean (SD), h[j]	7.1 (1.2)	6.8 (1.1)	6.8 (0.8)	.02
Sleep duration category[j]				
Short (<6 h)	20 (18.5%)	141 (22.0%)	24 (17.0%)	.003
Optimal (6-8 h)	65 (60.2%)	435 (67.8%)	107 (75.9%)	
Long (>8 h)	23 (21.3%)	66 (10.3%)	10 (7.1%)	
Sleep percentage[j]	88.1 (4.7)	90.1 (4.7)	91.2 (3.6)	<.001
Low sleep percentage[j] (<85%)	25 (23.1%)	76 (11.8%)	8 (5.7%)	<.001
Sleep fragmentation[j]	24.5 (8.9)	21.0 (7.7)	19.8 (7.1)	<.001
High fragmentation[j] (highest quartile)	40 (37.0%)	161 (25.1%)	21 (14.9%)	<.001
WASO[j], mean (SD), min	57.1 (23.2)	44.6 (22.6)	39.3 (16.6)	<.001
Prolonged WASO[j] (≥30 min)	98 (90.7%)	475 (74.0%)	96 (68.1%)	<.001

© 2026 Vu THT et al. *JAMA Network Open*.

Abbreviations: BMI, body mass index (calculated as weight in kilograms divided by height in 1 meters squared); CES-D, Center for Epidemiological Studies–Depression scale; WASO, wakefulness after sleep onset.

[a] *All characteristics were collected at the CARDIA year-35 core exam and Year-35 Sleep Ancillary Study, except for highest educational level.*

[b] *P-values for group comparisons based on χ^2 tests, F-tests, or Kruskal-Wallis tests.*

[c] *Highest educational level across CARDIA examinations.*

[d] *n= 855.*

[e] *n=858.*

[f] *n= 863.*

[g] *n=707.*

[h] *any use of cocaine, crack, amphetamines, methamphetamines, or heroin.*

[i] *any chronic diseases including heart diseases, stroke, hypertension, hypercholesterolemia, chronic obstructive pulmonary disease, or cancer.*

[j] *assessed using 7-day wrist actigraphy.*

© 2026 Vu THT et al. *JAMA Network Open*.

eTable 3: Analysis Sample Characteristics for Subjective Sleep Outcomes by Recent Cannabis Use Status

Characteristics[a]	Participants, No. (%)			P-value [b]
	Year-35 Recent Cannabis Use Status			
	Current use (n=126)	Former use (n=830)	Never use (n=172)	
Age, mean (SD), y	60.8 (3.6)	60.7 (3.5)	59.3 (3.9)	<.001
Sex				
Male	61 (48.4%)	317 (38.2%)	60 (34.9%)	0.04
Female	65 (51.6%)	513 (61.8%)	112 (65.1%)	
Race				
White	75 (59.5%)	495 (59.6%)	95 (55.2%)	0.56
Black	51 (40.5%)	335 (40.4%)	77 (44.8%)	
Study Site				
Birmingham, Alabama	10 (7.9%)	130 (15.7%)	64 (37.2%)	<.001
Chicago, Illinois	31 (24.6%)	256 (30.8%)	56 (32.6%)	
Minneapolis, Minnesota	44 (34.9%)	187 (22.5%)	29 (16.9%)	
Oakland, California	41 (32.5%)	257 (31.0%)	23 (13.4%)	
Highest educational level, mean (SD), y[c]	16.0 (2.6)	16.3 (2.5)	16.8 (2.6)	.02
Ever cigarette smokers	73 (57.9%)	305 (36.7%)	5 (2.9%)	<.001
Total exercise intensity score[d], mean (SD), exercise units	410.5 (342.3)	376.0 (300.6)	327.8 (256.7)	.05
Alcohol consumption[e], mL/day, median (IQR)	11.5 (0.0, 26.6)	4.8 (0.0, 14.8)	0.0 (0.0, 4.9)	<.001
Current drinkers[e]	92 (73.0%)	478 (57.6%)	60 (34.9%)	<.001
BMI, mean (SD)[f]	29.9 (6.5)	30.1 (7.2)	31.7 (7.8)	.03
Depression or CES-D scale score[g]	10.2 (8.1)	9.4 (8.0)	7.7 (7.0)	.03
Use of other substances[h]	28 (22.2%)	46 (5.5%)	1 (0.6%)	<.001
Use of sleep medicine	12 (9.5%)	47 (5.7%)	10 (5.8%)	.24
Any chronic diseases[i]	90 (71.4%)	588 (70.8%)	112 (65.1%)	.31
PSQI score[j]	7.1 (4.2)	6.3 (3.8)	6.0 (3.7)	.03
PSQI score >5[j]	75 (59.5%)	413 (49.8%)	78 (45.3%)	.05

Abbreviations: BMI, body mass index (calculated as weight in kilograms divided by height in 1 meters squared); CES-D, Center for Epidemiological Studies–Depression scale;

[a]All characteristics were collected at the CARDIA year-35 core exam and Year-35 Sleep Ancillary Study, except for highest educational level.

[b]P-values for group comparisons based on χ^2 tests, F-tests, or Kruskal-Wallis tests.

[c] Highest educational level across CARDIA examinations.

[d] n= 1112.

e n=1114.

[f] n= 1115.

[g] n=836.

[h]Any use of cocaine, crack, amphetamines, methamphetamines, or heroin.

[i] Any chronic diseases including heart diseases, stroke, hypertension, hypercholesterolemia, chronic obstructive pulmonary disease, or cancer.

[j]Pittsburgh Sleep Quality Index (PSQI) score assessed via self-reported questionnaires.

© 2026 Vu THT et al. *JAMA Network Open*.

eTable 4. Association[a] of Cumulative Lifetime Cannabis Use with Sleep Health Outcomes (Complete-Case Analyses)

Sleep Characteristics	Model 1			Model 2		
	β[b]	(95%CI)		β[b]	(95%CI)	
Complete-case sample						
**Actigraphy data (n= 681)**						
Sleep duration, h	0.004	-0.01	0.02	0.01	-0.01	0.02
Sleep percentage, %	**-0.18**	**-0.25**	**-0.12**	**-0.16**	**-0.22**	**-0.09**
Sleep fragmentation, %	**0.25**	**0.13**	**0.38**	**0.21**	**0.08**	**0.34**
WASO, min	**0.98**	**0.66**	**1.29**	**0.84**	**0.51**	**1.16**
**Self-reported data (n= 822)**						
Sleep quality or PSQI score	0.06	0.01	0.11	0.03	-0.03	0.08
Complete-case sample excluding participants with current cannabis use status						
**Actigraphy data (n= 584)**						
Sleep duration, h	-0.01	-0.04	0.03	0.002	-0.03	0.03
Sleep percentage, %	**-0.26**	**-0.38**	**-0.13**	**-0.22**	**-0.35**	**-0.09**
Sleep fragmentation, %	**0.30**	**0.06**	**0.53**	**0.27**	**0.02**	**0.52**
WASO, min	**1.34**	**0.75**	**1.93**	**1.22**	**0.59**	**1.85**
**Self-reported data (n= 706)**						
Sleep quality or PSQI score	0.08	-0.02	0.18	-0.01	-0.11	0.09

Abbreviations: PSQI, Pittsburgh Sleep Quality Index; WASO, wakefulness after sleep onset.

[a]Model adjusted for year-35 age, sex, race, study site, highest educational level, cumulative lifetime pack-years cigarette smoking, lifetime drink-years, recent use of other substances, physical activity, body mass index, sleep medication use, Center for Epidemiological Studies–Depression scale score, and comorbidity status.

[b]Expected change in sleep measures per one additional cannabis-year of exposure (1 cannabis-year = 365 days of cannabis use, derived from repeated CARDIA cannabis-use assessments).

© 2026 Vu THT et al. *JAMA Network Open*.

eTable 5. Association[a] of Cumulative Lifetime Cannabis Use With Sleep Health by Smoking Status

Sleep Characteristics	Never smoke cigarette			Ever smoke cigarette		
	β[b]	(95%CI)		β[b]	(95%CI)	
**Actigraphy data**						
Sleep duration, h	-0.01	-0.03	0.01	0.01	-0.01	0.03
Sleep percentage, %	**-0.20**	**-0.29**	**-0.12**	**-0.16**	**-0.25**	**-0.07**
Sleep fragmentation, %	**0.27**	**0.10**	**0.43**	**0.18**	**0.03**	**0.34**
WASO, min	**0.89**	**0.49**	**1.29**	**0.89**	**0.45**	**1.34**
**Self-reported data**						
Sleep quality or PSQI score	-0.01	-0.07	0.05	0.01	-0.06	0.08

Abbreviations: PSQI, Pittsburgh Sleep Quality Index; WASO, wakefulness after sleep onset.
[a]*Model adjusted for year-35 age, gender, race, study site, highest educational level, lifetime drink-years, recent use of other substances, physical activity, body mass index, sleep medication use, Center for Epidemiological Studies–Depression scale score, and comorbidity status, with missing covariate values imputed.*
[b]*Expected change in sleep measures per one additional cannabis-year of exposure (1 cannabis-year = 365 days of cannabis use, derived from repeated CARDIA cannabis-use assessments).*

© 2026 Vu THT et al. *JAMA Network Open*.

eTable 6. Association[a] of Cumulative Lifetime Cannabis Use with Sleep Health by Sex

Sleep Characteristics	Male			Female		
	**β[b]**	**(95%CI)**		**β[b]**	**(95%CI)**	
**Actigraphy data**						
Sleep duration, h	-0.01	-0.03	0.01	0.01	-0.02	0.03
Sleep percentage, %	**-0.16**	**-0.24**	**-0.08**	**-0.22**	**-0.31**	**-0.13**
Sleep fragmentation, %	**0.19**	**0.04**	**0.35**	**0.23**	**0.06**	**0.41**
WASO, min	0.67	0.28	1.05	1.23	0.78	1.68
**Self-reported data**						
Sleep quality or PSQI score	0.04	-0.01	0.09	-0.05	-0.12	0.03

Abbreviations: PSQI, Pittsburgh Sleep Quality Index; WASO, wakefulness after sleep onset.

[a]*Model adjusted for year-35 age, race, study site, highest educational level, cumulative lifetime pack-years cigarette smoking, lifetime drink-years, recent use of other substances, physical activity, body mass index, sleep medication use, Center for Epidemiological Studies–Depression scale score, and comorbidity status, with missing covariate values imputed.*

[b]*Expected change in sleep measures per one additional cannabis-year of exposure (1 cannabis-year = 365 days of cannabis use, derived from repeated CARDIA cannabis-use assessments).*

© 2026 Vu THT et al. *JAMA Network Open*.

eTable 7. Association[a] of Cumulative Lifetime Cannabis Use With Sleep Health by Drinking Status

Sleep Characteristics	Current Drinkers			Non-Current Drinkers		
	**β[b]**	**(95%CI)**		**β[b]**	**(95%CI)**	
**Actigraphy data**						
Sleep duration, h	-0.01	-0.02	0.01	0.01	-0.02	0.04
Sleep percentage, %	**-0.15**	**-0.22**	**-0.08**	**-0.33**	**-0.44**	**-0.22**
Sleep fragmentation, %	**0.18**	**0.04**	**0.32**	**0.36**	**0.15**	**0.56**
WASO, min	0.69	0.35	1.02	**1.73**	**1.18**	**2.28**
**Self-reported data**						
Sleep quality or PSQI score	0.04	-0.01	0.09	-0.02	-0.09	0.06

Abbreviations: PSQI, Pittsburgh Sleep Quality Index; WASO, wakefulness after sleep onset.

[a]*Model adjusted for year-35 age, gender, race, study site, highest educational level, cumulative lifetime pack-years cigarette smoking, recent use of other substances, physical activity, body mass index, sleep medication use, Center for Epidemiological Studies–Depression scale score, and comorbidity status, with missing covariate values imputed.*

[b]*Expected change in sleep measures per one additional cannabis-year of exposure (1 cannabis-year = 365 days of cannabis use, derived from repeated CARDIA cannabis-use assessments).*

© 2026 Vu THT et al. *JAMA Network Open*.

eTable 8. Association[a] of Cumulative Lifetime Cannabis Use With Sleep Health by Recent Use of Other Substances

	Recent Use of Other Substances					
**Sleep Characteristics**	**Recent Users**			**Non-Recent Users**		
	**β[b]**	**(95%CI)**		**β[b]**	**(95%CI)**	
**Actigraphy data**						
Sleep duration, h	0.01	-0.06	0.08	-0.004	-0.02	0.01
Sleep percentage, %	-0.001	-0.23	0.23	**-0.21**	**-0.27**	**-0.14**
Sleep fragmentation, %	-0.10	-0.51	0.31	**0.24**	**0.12**	**0.36**
WASO, min	0.05	-0.95	1.05	**0.96**	**0.65**	**1.26**
**Self-reported data**						
Sleep quality or PSQI score	-0.02	-0.14	0.10	0.004	-0.04	0.05

Abbreviations: PSQI, Pittsburgh Sleep Quality Index; WASO, wakefulness after sleep onset.

[a]*Model adjusted for year-35 age, gender, race, study site, highest educational level, cumulative lifetime pack-years cigarette smoking, lifetime drink-years, physical activity, body mass index, sleep medication use, Center for Epidemiological Studies–Depression scale score, and comorbidity status, with missing covariate values imputed.*

[b]*Expected change in sleep measures per one additional cannabis-year of exposure (1 cannabis-year = 365 days of cannabis use, derived from repeated CARDIA cannabis-use assessments).*

© 2026 Vu THT et al. *JAMA Network Open*.

eAppendix: Missing Data Imputation and Computation of Cumulative Lifetime Cannabis Use

3.1.1: Self-reported Questions

At each CARDIA examination, participants self-reported their cannabis use through the following questions: "*Have you ever used marijuana (cannabis)*?" Those who answered yes were then asked "*During the last 30 days, on how many days did you use marijuana (cannabis)?* " And "*About how many times in your lifetime have you used marijuana (cannabis)?* " These questions were used to compute cumulative lifetime cannabis exposure.

3.1.2. Missing Cannabis-Use Data Imputation

The study included only participants with sufficient cannabis-use data, defined as having cannabis-use data from at least six examinations, or from both the Year-35 examination and at least one other examination.

Missing cannabis data were reviewed to identify patterns of missingness before imputation. Among 1,266 participants with valid objective (n=891) or subjective sleep data (n=1,128), 761 participants (60.1%) had complete cannabis-use data across all 10 examinations. Of the remaining 505 participants with missing cannabis-use data at one or more examinations, 81% had missing data at no more than 2 of the 10 examination visits. Missingness was predominantly non-monotone, indicating intermittent missing cannabis-use assessments across study visits. Only 50 participants (4%) had long missing intervals (i.e., missing > 2 consecutive examinations); however, these participants generally reported either never using cannabis or discontinuing cannabis use at examinations before and after the missing interval. Thus, imputation of these intervals did not result in additional cannabis-use days being accrued.

Missing values at the baseline examination were imputed by carrying the first available post-baseline value backward, whereas missing values at Year 35 examination were imputed by carrying the last available pre-Year 35 value forward.

Cannabis-use data were then expanded from examination-level observation to annual observations spanning Years 0 through 35. Annual cannabis-use values during intervals between examinations were estimated under the assumption that the reported past 30-day frequency at each examination represented the participant's average use during the surrounding interval. Years before the midpoint were assigned the value from the preceding examination, whereas years after the midpoint were assigned the value from the subsequent examination. When an interval contained an odd number of annual observations, the midpoint year was assigned the value from the preceding examination.

3.1.3. Computation of Cumulative Lifetime Cannabis Use

Reported lifetime use at baseline was used to estimate cannabis exposure before study entry. Cumulative lifetime cannabis use was calculated by summing estimated pre-baseline exposure and annual cannabis exposure accrued during follow-up. At each follow-up examination, if a participant's self-reported lifetime use exceeded the cumulative estimate derived from the interval calculations, the cumulative estimate was updated to the higher self-reported lifetime value. Cumulative cannabis exposure at Year 35 was expressed as cannabis-years, where one cannabis-year was defined as 365 days of cannabis use.

Box 1 provides a worked example from one participant, illustrating the variables used in the computation of cumulative lifetime cannabis use:

exam: CARDIA examinations with intervals of 2-5 year between visits.
year: Annual follow-up year (Year 0-35)

© 2026 Vu THT et al. *JAMA Network Open*.

mj30: Self-reported number of days of using cannabis during the past 30 days.
life: Self-reported lifetime cannabis use.
mj30_imp: Imputed *mj30* variable after applying the missing data imputation algorithm.
life_imp: Imputed *life* variable after applying the missing data imputation algorithm.
cum_days: Running cumulative number of days of cannabis use accrued from baseline through each follow-up year.
cum_yrs: Cumulative cannabis exposure expressed as cannabis-years (*cum_days* /365).

© 2026 Vu THT et al. *JAMA Network Open*.

Box 1. Example of computing cannabis-years for one participant:

exam	*year*	*mj30*	*life*	*mj30_imp*	*life_imp*	*cum_days*	*cum_yrs*
Baseline	0	5	300	5	300	300	0.82
	1	.	.	5	300	360.83	0.99
Year 2	2	5	300	5	300	421.67	1.16
	3	.	.	5	300	482.5	1.32
	4	.	.	2	300	506.83	1.39
Year 5	5	2	300	2	300	531.17	1.46
	6	.	.	2	300	555.5	1.52
Year 7	7	2	300	2	300	579.83	1.59
	8	.	.	2	300	604.17	1.66
	9	.	.	2	700	628.5	1.72
Year 10	10	2	300	2	300	652.83	1.79
	11	.	.	2	300	677.17	1.86
	12	.	.	2	300	701.5	1.92
	13	.	.	15	700	884	2.42
	14	.	.	15	700	1066.5	2.92
Year 15	15	15	700	15	700	1249	3.42
	16	.	.	15	700	1431.5	3.92
	17	.	.	15	700	1614	4.42
	18	.	.	5	700	1674.84	4.59
	19	.	.	5	700	1735.67	4.76
Year 20	20	5	700	5	700	1796.5	4.92
	21	.	.	5	700	1857.34	5.09
	22	.	.	5	700	1918.17	5.26
	23	.	.	0	700	1918.17	5.26
	24	.	.	0	700	1918.17	5.26
Year 25	25	0	700	0	700	1918.17	5.26
	26	.	.	0	700	1918.17	5.26
	27	.	.	0	700	1918.17	5.26
	28	.	.	0	700	1918.17	5.26
	29	.	.	0	700	1918.17	5.26
Year 30	30	0	Missing	**0**	**(midpoint) 700**	1918.17	5.26
	31	.	.	*0*	*700*	1918.17	5.26
	32	.	.	*0*	*700*	1918.17	5.26
	33	.	.	*0*	*700*	1918.17	5.26
	34	.	.	*0*	*700*	1918.17	5.26
Year 35	35	0	700	*0*	*700*	1918.17	5.26

© 2026 Vu THT et al. *JAMA Network Open*.

According to this example, the participant had cannabis-use data for 9 of the 10 examination years, with only the Year 30 assessment missing.

At baseline (Year 0), the participant reported lifetime cannabis use of 100 to 499 times, which was converted to 300 lifetime uses (*life=300*). This value was used to estimate cannabis exposure accrued before study entry, resulting in *cum_days* =300 and *cum-yrs*=300/365=0.82 cannabis-years.

The participant also reported using cannabis on 5 days during the past 30 days (*mj30*=5). Because the interval between Year 0 and Year 2 contained one annual observation (Year 1), the midpoint rule assigned the Year 0 value to Year 1 (*mj30_imp* = 5). Estimated cannabis use during that year was calculated as 5 × (365/30) = 60.83 days. Thus, *cum_days* =300+60.83=360.83 and *cum_yrs*=360.83/365=0.99 at Year 1. This procedure was repeated for each subsequent follow-up year.

Between Year 2 and Year 5, the participant reported *mj30*=5 at Year 2 and *mj30*=2 at Year 5. The interval contained two annual observations (Years 3 and 4), therefore, Year 3 was assigned the Year 2 value (*mj30_imp* = 5), whereas Year 4 was assigned the Year 5 value (*mj30_imp* = 2).

The Year-30 data was missing. Consequently, the interval between Year 25 and 35 contained nine annual observations (Year 26-34). Because the interval contained an odd number of annual observations, Years 26-30 (including the midpoint year) were assigned from the preceding examination (Year 25), whereas Years 31-34 were assigned values from the subsequent examination (Year 35). In this example, cannabis-use values were identical at Years 25 and 35 (*mj30*=0 and *life*=700), resulting in identical imputed values across the interval.

Participants subsequently accrued cumulative lifetime cannabis exposure over follow-up, expressed as *cum_days* and *cum_yrs* through Year 35. For this participant, the final cumulative exposure was 5.26 cannabis-years, corresponding to 1918.17 estimated days of cannabis use. This imputation and cumulative exposure calculation approach has been used in several of our previous publications.[1-6]

© 2026 Vu THT et al. *JAMA Network Open*.

eReferences

1. Jakob J, Schwerdtel F, Sidney S, et al. Associations of cannabis use and body mass index-The Coronary Artery Risk Development in Young Adults (CARDIA) study. *Eur J Intern Med*. Nov 2024;129:41-47. doi:10.1016/j.ejim.2024.07.007
2. Pletcher MJ, Vittinghoff E, Kalhan R, et al. Association between marijuana exposure and pulmonary function over 20 years. *JAMA*. Jan 11 2012;307(2):173-81. doi:10.1001/jama.2011.1961
3. Auer R, Vittinghoff E, Yaffe K, et al. Association Between Lifetime Marijuana Use and Cognitive Function in Middle Age: The Coronary Artery Risk Development in Young Adults (CARDIA) Study. *JAMA Internal Medicine*. 2016;176(3):352-361. doi:10.1001/jamainternmed.2015.7841
4. Reis JP, Auer R, Bancks MP, et al. Cumulative Lifetime Marijuana Use and Incident Cardiovascular Disease in Middle Age: The Coronary Artery Risk Development in Young Adults (CARDIA) Study. *Am J Public Health*. Apr 2017;107(4):601-606. doi:10.2105/AJPH.2017.303654
5. Corroon J, Bradley R, Grant I, et al. Lifetime Cannabis Use and Incident Hypertension: The Coronary Artery Risk Development in Young Adults (CARDIA) Study. *Hypertension*. Oct 2025;82(10):1641-1652. doi:10.1161/HYPERTENSIONAHA.125.25005
6. Pasquier B, Yaffe K, Levine DA, et al. Sex Differences in the Association Between Cumulative Use of Cannabis and Cognitive Function in Middle Age: The Coronary Artery Risk Development in Young Adults Study. *Cannabis Cannabinoid Res*. Aug 2024;9(4):e1142-e1158. doi:10.1089/can.2022.0343

© 2026 Vu THT et al. *JAMA Network Open*.
