# Supplementary material for: Recent and Cumulative Cannabis Use and Sleep in Middle-Aged Adults
Source: JAMA Netw Open. 2026 Oct 9;9(10):e2638303. doi: 10.1001/jamanetworkopen.2026.38303 (PMC13653285; doi:10.1001/jamanetworkopen.2026.38303)
Supplement: Supplementary file 2 [file jamanetwopen-e2638303-s002.pdf]

Data Sharing Statement

Vu. Recent and Cumulative Cannabis Use and Sleep in Middle-Aged Adults. *JAMA Netw Open*. Published October 09, 2026. doi:10.1001/jamanetworkopen.2026.38303

Data
Data available: No

Additional Information
Explanation for why data not available: Data from CARDIA and CARDIA Sleep are not publicly available. The study team does not have authorization to share these data. Access to the data is governed by data use agreements by the CARDIA Coordinating Center. More details about the CARDIA Study and associated documentation are available at: https://www.cardia.dopm.uab.edu/. CARDIA also deposits data into the NHBLI's Biologic Specimen and Data Repository Information Coordinating Center (BioLINCC) website (https://biolincc.nhlbi.nih.gov/home). Interested investigators are encouraged to review protocols and data available on CARDIA's website and review procedures for applying for access.
